# Supplementary material for: Antibacterial Activity of Non-Cytotoxic, Amino Acid-Modified Polycationic Dendrimers against Pseudomonas aeruginosa and Other Non-Fermenting Gram-Negative Bacteria
Source: Polymers (Basel). 2020 Aug 13;12(8):1818. doi: 10.3390/polym12081818 (PMC7464783; doi:10.3390/polym12081818)
Supplement: Supplementary file 1 [file polymers-12-01818-s001.pdf]

Supplementary Materials

Antibacterial activity of non-cytotoxic, amino acid-modified polycationic dendrimers against *Pseudomonas aeruginosa* and other non-fermenting Gram-negative bacteria

Anna Maria Schito [1], and Silvana Alfei [2,*]

[1] Department of Surgical Sciences and Integrated Diagnostics (DISC), University of Genoa, Viale Benedetto XV, 6, I-16132 Genova, Italy, amschitonige.it
[2] Department of Pharmacy (DiFAR), University of Genoa, Viale Cembrano 4, I-16148, Genova, Italy, alfei@difar.unige.it
* Correspondence: alfei@difar.unige.it; Tel.: +39-010-335-2296 (S.A.)

Table of Content

Part S1. Dendrimers

Section S1. Molecular Weight and buffer capacity of dendrimers G5K, G5H and G5HK

Table S1. Molecular Weights of dendrimers G5K, G5H and G5HK estimated by ^{1}H NMR and from titration with $HClO_4$.
Figure S1. Buffer capacity (β = dCa/dpH) of dendrimers under study compared with b-PEI and G4-PAMAMs.
Figure S2. Average buffer capacity ($\overline{\beta}$ = dV(mL)/dpH(1)) of dendrimers under study compared with b-PEI and G4-PAMAMs.
Table S2. β (pH around 6) and $\overline{\beta}$ (pH = 4.5-7.5) of dendrimers G5K, G5H and G5HK from potentiometric titrations.

Section S2. Results from binding test with plasmid DNA and from experiments of some G5Ds penetration in human Hela cells

Figure S3. Results from binding test with plasmid DNA for G5HK (A6), G5H (A7) and G5K (A8).
Figure S4. Images obtained with a double fluorescence microscope of Hela cells in incomplete culture medium (a) and in complete medium (b) from experiments of penetration of some G5Ds including G5K.

Part S2. Microbiology

Section S3. Strains susceptibilities

Table S3. Antimicrobial susceptibility patterns of the 9 *Pseudomonas aeruginosa* (strain 249 is highly mucous) of *Pseudomonas fluorescens* (strain 263) and of *Pseudomonas putida* (*strain 262)* isolates employed in the study.
Table S4. Antimicrobial susceptibility patterns of the 4 *Acinetobacter baumannii* strains and of the *Acinetobacter pittii* (strain 272) isolates employed in the study.
Table S5. Antimicrobial susceptibility patterns of the 4 Stenotrophomonas maltophilia strains employed in the study.
Table S6. Antimicrobial susceptibility patterns of the 2 *Klebsiella pneumoniae* strains employed in the study.
Table S7. Antimicrobial susceptibility patterns of the 2 *Escherichia coli* (strains 123 and 133) and *Proteus mirabilis* (strain 155) isolates employed in the study.
Table S8. Antimicrobial susceptibility patterns of the 4 *Enterococcus* isolated employed in the study.
Table S9. Antimicrobial susceptibility patterns of the 4 *Staphylococcus* isolates employed in the study.

Section S1

Table S1. Molecular Weights of dendrimers G5K, G5H and G5HK estimated by ^{1}H NMR and from titration with $HClO_4$ [1, 2].

Dendrimer	N *	MW (Calcd.) ¹	MW (obs.) ²
G5K	192	30849	**28966**
G5H	192	31085	**29141**
G5K(50)H(46)	192	30637	**30592**

* number of peripheral basic groups as determined by NMR; ¹ estimated by ^{1}H NMR; ² obtained by volumetric titration.

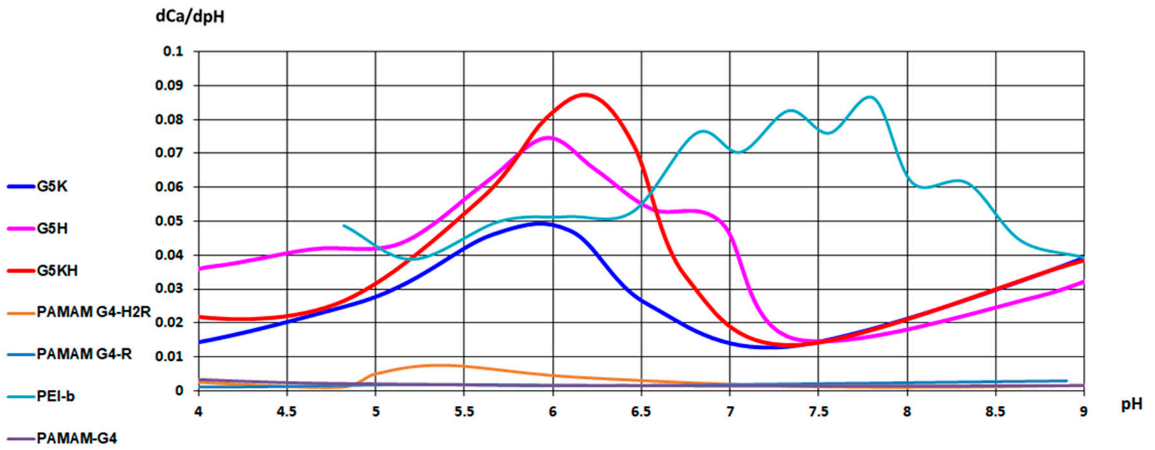

Figure S1. Buffer capacity (β = dCa/dpH) of dendrimers under study compared with b-PEI and G4-PAMAMs [1].

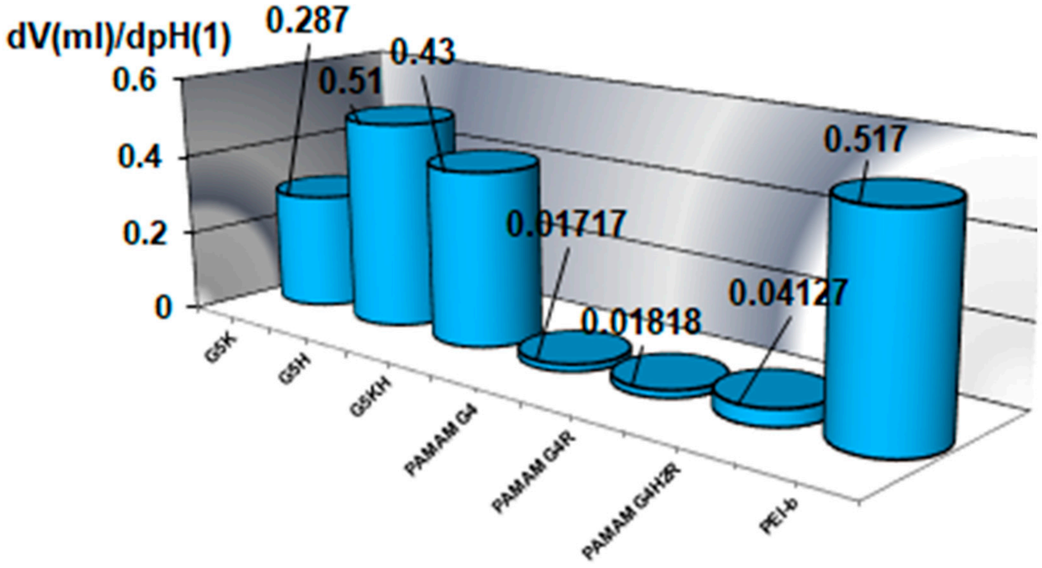

Figure S2. Average buffer capacity ($\overline{\beta}$ = dV(mL)/dpH(1)) of dendrimers under study compared with b-PEI and G4-PAMAMs [1].

Table S2. β (pH around 6) and $\overline{\beta}$ (pH = 4.5-7.5) of dendrimers G5K, G5H and G5HK from potentiometric titrations [1].

**Dendrimer**	**N** *	β	$\overline{\beta}$ [1]
G5K	192	0.0472	0.287
G5H	192	**0.0653**	0.510
G5K(50)H(46)	192	**0.0870**	0.430
**b-PEI** [2]	-	**0.0760**	**0.517**
**G4-PAMAM** [3]	-	0.0014	0.017
**G4-PAMAM-Arg** [4]	-	0.0015	0.018
**G4-PAMAM-HisHisArg** [5]	-	0.0038	0.041

* number of peripheral basic groups as determined by NMR; [1] calculated for three degree of freedom; [2] non-dendrimeric branched structure; [3] fourth generation PAMAM; [4] G4 PAMAM containing arginine; [5] G4-PAMAM containing the His-His-Arg sequence.

Section S2. Results from binding test with plasmid DNA and from experiments of penetration in human Hela cells performed by using some G5Ds

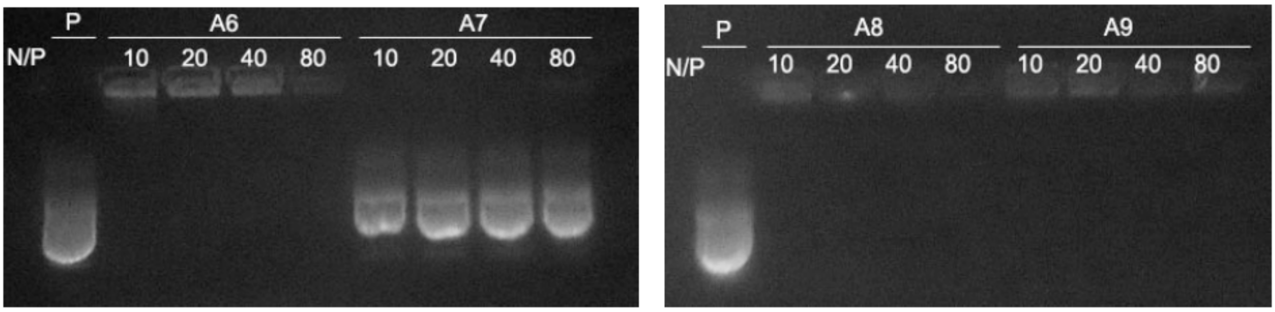

Figure S3. Results from binding test with plasmid DNA for G5HK (A6), G5H (A7) and G5K (A8) [1].

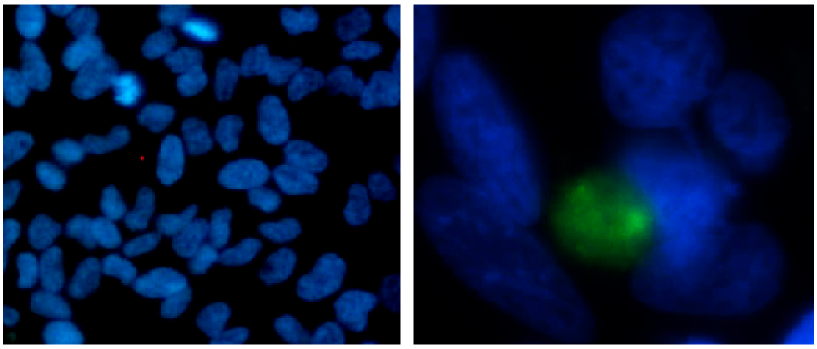

a b

Figure S4. Images obtained with a double fluorescence microscope of Hela cells in incomplete culture medium (a) and in complete medium (b) from experiments of penetration of some G5Ds including G5K [1].

Part S2. Microbiology

Section 3. Strains susceptibilities

Table S3. Antimicrobial susceptibility patterns of the 9 *Pseudomonas aeruginosa* (strain 249 is highly mucous) of *Pseudomonas fluorescens* (strain 263) and of *Pseudomonas putida* (strain 262*)* employed in the study.

	**209**	**230**	**247**	**248**	**249**	**253**	**256**	**259**	**265**	**262**	**263**
Amikacin	R	S	S	S	S	S	S	R	S	S	S
Ciprofloxacin	S	R	R	R	S	R	S	R	I	S	S
Cefepime	S	S	I	R	R	R	I	R	I	R	I
Ceftazidime	S	S	I	R	S	I	R	R	R	I	S
Ceftazidime/Avibactam	S	S	S	S	S	S	S	R	S	S	S
Gentamicin	S	S	S	S	S	S	S	S	S	S	S
Meropenem	S	S	R	S	S	S	R	R	S	S	S
Piperacillin/Tazobactm	R	S	I	R	S	I	R	R	I	R	S
Colistin	S	S	S	S	S	S	S	S	R	S	S

Table S4. Antimicrobial susceptibility patterns of the 4 *Acinetobacter baumannii* and of the *Acinetobacter pittii* (strain 272) employed in the study.

	**24**	**25**	**26**	**27**	**272**
Amikacin	R	R	S	S	S
Ciprofloxacin	I	R	S	R	S
Gentamicin	R	R	R	S	S
Meropenem	S	R	R	S	S
Trimethoprim/Sulfamethoxazole	S	S	S	S	S
Colistin	S	S	S	S	S

Table S5. Antimicrobial susceptibility patterns of the 4 *Stenotrophomonas maltophilia* employed in the study.

	**11**	**16**	**18**	**19**
Trimetoprim/Sulfamethoxazole	S	I	S	S
Ciprofloxacin	-	-	-	-

Table S6. Antimicrobial susceptibility patterns of the 2 *Klebsiella pneumoniae* employed in the study.

	**236**	**237**
Amikacin	R	S
Amoxicillin/Clavulanate	R	S
Cefepime	R	R
Cefotaxime	R	R
Ciprofloxacin	R	I
Colistin	S	S
Ertapenem	R	S
Gentamicin	R	S
Meropenem	R	S
Piperacillin/Tazobactam	R	R
Trimethoprim/Sulfamethoxazole	S	S

Table S7. Antimicrobial susceptibility patterns of the 2 *Escherichia coli* (strains 123 and 133) and *Proteus mirabilis* (strain 155) employed in the study.

	***E. coli* 123**	***E. coli* 133**	***P. mirabilis* 155**
Amikacin	S	S	S
Ciprofloxacin	R	R	R
Amoxicillin/Clavulanate	S	S	S
Ertapenem	S	S	S
Cefepime	S	R	S
Cefotaxime	S	R	S
Ceftazidime	S	R	S
Colistin	S	S	S
Fosfomycin	S	S	S
Gentamicin	S	S	S
Imipenem	S	S	S
Meropenem	S	S	S
Nitrofurantoin	S	S	S
Piperacillin/Tazobactam	S	S	S
Trimethoprim/Sulfamethoxazole	R	S	R

Table S8. Antimicrobial susceptibility patterns of the 4 *Enterococcus* isolated employed in the study.

	***E. faecalis* 120**	***E. faecalis* 124**	***E. faecium* 118**	***E. faecium* 127**
Ampicillin	R	S	R	R
Ampicillin/Sulbactam	I	S	R	R
Cefuroxime	R	R	R	R
Clindamicin	R	R	R	R
Erythromycin	R	R	R	R
Gentamicin	R	S	S	R
Imipenem	R	S	R	R
Levofloxacin	R	R	R	R
Linezolid	S	S	S	S
Nitrofurantoin	S	S	S	S
Quinupristin/Dalfopristin	R	R	S	S
Teicoplanin	R	S	R	S
Tigecycline	S	S	S	S
Trimethoprim/ Sulfamethoxazole	R	R	R	R
Vancomicin	R	S	R	S

Table S9. Antimicrobial susceptibility patterns of the 4 *Staphylococcus* isolates employed in the study.

	S. aureus 118	***S. aureus 119***	***S. epidermidis 197***	***S. epidermidis 199***
Ciprofloxacin	R	S	R	R
Clindamicin	S	S	R	R
Daptomicin	S	S	S	S
Doxycycline	S	S	S	S
Gentamicin	S	S	R	R
Levofloxacin	R	S	R	S
Linezolid	S	S	S	S
Moxifloxacin	R	S	R	S
Oxacillin	R	S	R	S
Rifampicin	S	S	R	S
Tetracycline	S	R	I	R
Tigecycline	S	S	S	S
Trimethoprim/Sulfamethoxazole	S	S	I	S
Vancomicin	S	S	S	S

References

1. Alfei, S.; Castellaro, S.; Taptue GB. Synthesis and NMR characterization of dendrimers based on 2,2-*bis*-(hydroxymethyl)-propanoic acid (*bis*-HMPA) containing peripheral amino acid residues for gene transfection. *Org. Commun.* **2017**, *10*, 144-77.
2. Vogel, A. I. Part III. Quantitative organic analysis. In *Elementary Practical Organic Chemistry*, London: Longman, 1958; pp. 702-705.
